# Renal Angina Index as a Predictor of Acute Kidney Injury in Critically Ill Children: A Prospective Observational Study

**DOI:** 10.7759/cureus.110338

**Published:** 2026-06-06

**Authors:** Umesh Pandwar, Nitesh Upadhyay, Sumit Saad, Mohd Shahid, Navindana, Manjusha Goel

**Affiliations:** 1 Paediatrics, Gandhi Medical College, Bhopal, IND

**Keywords:** acute kidney injury, kdigo, kidney, pediatric intensive care, renal angina index

## Abstract

Objective

This study aimed to evaluate the effectiveness of the Renal Angina Index (RAI) in predicting acute kidney injury (AKI) among pediatric ICU (PICU) patients.

Methods

This prospective observational study, conducted at Gandhi Medical College, Bhopal, from September 2022 to February 2024, included 300 PICU patients aged one month to 13 years. RAI was calculated 24 hours post-admission, and its correlation with the development of AKI after 24 hours of admission was analyzed using SPSS Statistics version 25 (IBM Corp., Armonk, NY).

Results

The mean patient age was 36.16 months (standard deviation (SD): 15.08); 50.7% were male. AKI occurred in 5% of patients. Mortality increased significantly across RAI categories: 0% (low-risk), 7.5% (moderate-risk), and 68.8% (high-risk) (p < 0.001). RAI at 24 hours demonstrated excellent discriminatory ability with an area under the curve (AUC) of 0.87; 95% confidence interval (CI): 0.80-0.94. At a threshold ≥ 8, RAI showed 81.8% sensitivity and 97.2% specificity, with a 75.0% positive predictive value (PPV). Strong associations existed between RAI and vasopressor support, PRISM (Pediatric Risk of Mortality) scores, and KDIGO (Kidney Disease: Improving Global Outcomes)-documented AKI (all p < 0.001).

Conclusions

RAI can predict AKI in critically ill pediatric patients, enabling early intervention. The 24-hour assessment provides valuable prognostic information for patient risk stratification.

## Introduction

Acute kidney injury (AKI) occurs in approximately 30% of children admitted to pediatric ICUs (PICU), and it contributes significantly to morbidity and mortality [1]. The condition poses significant clinical challenges, as many medications require dose adjustment due to renal elimination pathways [2]. Mortality rates associated with AKI remain concerning, with reported deaths of 23.9% in adults and 13.8% in pediatric patients [3]. The severity of AKI is directly correlated with mortality risk, emphasizing the importance of early prediction and timely intervention [4,5].

Although the KDIGO (Kidney Disease: Improving Global Outcomes) guidelines classify AKI based on serum creatinine and urine output, serum creatinine has notable limitations. It rises late in the disease course, lacks specificity for intrinsic kidney injury, and can be influenced by non-renal factors, including autoregulation and reduced glomerular filtration [6,7]. These limitations have prompted the search for more sensitive early prediction tools.

The Renal Angina Index (RAI) was developed to predict AKI in critically ill patients by combining patient risk factors with early signs of kidney injury [8]. The term is inspired by angina pectoris in cardiology, serving as an early warning sign of organ stress or damage. Preliminary studies suggest that RAI may enable earlier AKI detection in high-risk populations [9,10]. Nevertheless, further validation across diverse populations and settings is needed.

This study primarily aims to evaluate the role of RAI in predicting subsequent KDIGO-defined AKI among pediatric patients admitted to a tertiary care PICU in central India. Specifically, we sought to assess the association between RAI scores and the development of severe AKI in resource-limited settings.

## Materials and methods

Study design, setting, and ethical considerations

This prospective observational study was conducted in the PICU at Gandhi Medical College, Bhopal, from September 2022 to February 2024. The study was approved by the Institutional Ethics Committee, Gandhi Medical College, Bhopal. Written informed consent was obtained from parents or guardians of all participants.

Study population

Children aged one month to 13 years admitted to the PICU were included. Exclusion criteria included (i) chronic diseases or pre-existing organ failure, (ii) PICU admission within the preceding 90 days, and (iii) age younger than one month or older than 13 years. The sample size was calculated using the formula 4PQ/d² based on preliminary data, yielding 195 patients.

Data collection

Demographic information, clinical history, and laboratory findings were recorded using a structured proforma. Key parameters assessed included estimated creatinine clearance (eCrCl), fluid overload percentage, and RAI. Baseline investigations, including serum creatinine, were performed at admission after a detailed history and physical examination.

RAI calculation

**Figure 1:**
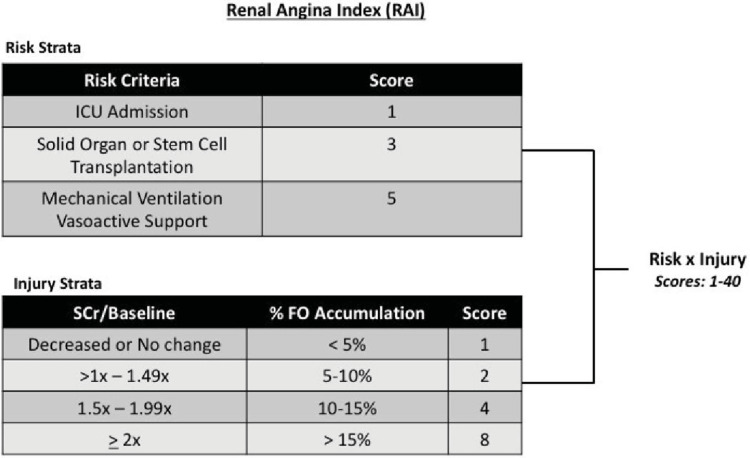
Renal Angina Index calculation ICU: intensive care unit; sCr: serum creatinine; FO: fluid overload

RAI was calculated 24 hours post-admission using risk stratification (based on admission diagnosis and comorbidities) and injury scores (based on changes in creatinine and fluid overload) (Figure 1).

RAI was considered positive if the score exceeded 8. Patients were monitored for fluid overload, and RAI scores were calculated at multiple timepoints during PICU stay.

AKI definition

AKI was classified according to KDIGO criteria based on serum creatinine elevation and urine output changes. Patients were followed throughout their PICU stay for AKI development and outcomes.

Statistical analysis

Data were analyzed using Microsoft Excel (Microsoft Corporation, Redmond, WA) and IBM SPSS Statistics version 25 (IBM Corp., Armonk, NY). Descriptive statistics included means ± standard deviations (SD) for continuous variables and frequencies with percentages for categorical variables. Chi-square tests, Fisher's exact test, and ANOVA were applied as appropriate. Receiver operating characteristic (ROC) curve analysis was used to assess RAI's discriminatory ability. The area under the curve (AUC) with 95% confidence intervals (CIs) was calculated, and sensitivity, specificity, positive predictive value (PPV), and negative predictive value (NPV) were determined for different RAI thresholds. Statistical significance was set at p < 0.05.

## Results

The study included 198 PICU-admitted patients from a total of 300 pediatric admissions, with a mean age of 36.16 (SD: 15.08) months. Among them, 105 (53%) were male. Primary admission diagnoses included gastrointestinal disorders (26.8%), respiratory disorders (28.3%), neurological disorders (17.8%), hematological disorders (8.58%), shock (8%), cardiovascular disorders (8%), and endocrine disorders (1.7%). Thirty-seven patients (18.7%) were malnourished. Mechanical ventilation was required in 33 patients (16.7%), and 16 patients (8%) received vasopressor support. Overall mortality was 7.07% (14 patients), and 184 patients (92.9%) were discharged.

RAI distribution and clinical associations

Among 198 patients, 142 (47.3%) had low-risk RAI (score 2), 40 (13.3%) had moderate-risk RAI (scores 5-6), and 16 (5.3%) had high-risk RAI (scores 8-10). Demographic factors, such as age group and gender, were not significantly associated with RAI scores (p = 0.173 and p = 0.892, respectively). Most affected organ systems did not show a statistically significant relationship with RAI categorization, though cardiovascular involvement approached significance (p = 0.078) (Table 1).

**Table 1:** Association of RAI with other clinical factors RAI: Renal Angina Index; CNS: central nervous system; PRISM: Pediatric Risk of Mortality; PICU: pediatric intensive care unit; KDIGO: Kidney Disease: Improving Global Outcomes; SD: standard deviation

Clinical factor		RAI = 2 (low risk), n = 142	RAI = 5-6 (moderate risk), n = 40	RAI = 8-10 (high risk), n = 16	χ^2^	P-value
Age group, n (%)						
1 month, 2 years		86 (60.6%)	23 (57.5%)	10 (62.5%)	6.40	0.173
> 2-5 years		34 (23.9%)	7 (17.5%)	3 (18.8%)		
≥ 5 years		22 (15.5%)	10 (25.0%)	3 (18.8%)		
Gender, n (%)						
Male		76 (53.5%)	20 (50.0%)	9 (56.3%)	0.23	0.892
Female		66 (46.5%)	20 (50.0%)	7 (43.8%)		
System affected, n (%)						
Respiratory		45 (31.7%)	7 (17.5%)	4 (25.0%)		0.149
Gastrointestinal		35 (24.6%)	14 (35.0%)	4 (25.0%)		0.390
CNS		27 (19.0%)	7 (17.5%)	5 (31.3%)		0.420
Cardiovascular		8 (5.6%)	5 (12.5%)	3 (18.8%)		0.078
Hematological		12 (8.5%)	4 (10.0%)	1 (6.3%)		0.875
Clinical parameters, n (%)						
Vasopressor support		0 (0%)	6 (15.0%)	10 (62.5%)	88.52	< 0.001
Malnourished		22 (15.5%)	11 (27.5%)	4 (25.0%)	3.97	0.137
PRISM score ≥ 10 at admission		0 (0%)	13 (32.5%)	16 (100%)	154.36	< 0.001
48-96-hour PICU stay		0 (0%)	10 (25.0%)	6 (37.5%)	55.47	< 0.001
Length of stay > 7 days		27 (19.0%)	11 (27.5%)	4 (25.0%)	1.67	0.434
Nephrotoxic medications		2 (1.4%)	4 (10.0%)	3 (18.8%)	11.62	< 0.001
KDIGO stage, n (%)						
Documented KDIGO at 24h		0 (0%)	5 (12.5%)	10 (62.5%)	102.41	< 0.001
Documented KDIGO at 48h		0 (0%)	5 (12.5%)	10 (62.5%)	102.41	< 0.001
Outcomes						
Mortality, n (%)		0 (0%)	3 (7.5%)	11 (68.8%)	131.78	< 0.001
Mean PRISM score at admission (± SD)		1.30 (± 1.79)	5.75 (± 4.81)	13.94 (± 5.26)	154.36	< 0.001

Strong associations emerged between RAI categories and several clinical parameters. Vasopressor support showed a clear gradient: 0% in low-risk, 15.0% in moderate-risk, and 62.5% in high-risk patients (p < 0.001). PRISM scores ≥ 10 were absent in low-risk patients, present in 32.5% of moderate-risk patients, and universal (100%) in high-risk patients (p < 0.001). Extended PICU stays (48-96 hours) and nephrotoxic medication use increased significantly with rising RAI categories (both p < 0.001).

RAI and AKI

KDIGO-documented AKI appeared in none of the low-risk patients, 12.5% of moderate-risk patients, and 62.5% of high-risk patients (p < 0.001). Mortality demonstrated a powerful relationship with RAI category: 0% in low-risk, 7.5% in moderate-risk, and 68.8% in high-risk patients (p < 0.001). Mean PRISM scores at admission increased stepwise: 1.30 (SD: 1.79) in low-risk, 5.75 (SD: 4.81) in moderate-risk, and 13.94 (SD: 5.26) in high-risk patients (p < 0.001) (Table 2).

**Table 2:** Descriptive overview of pediatric patients in PICU PICU: pediatric intensive care unit; SD: standard deviation

Parameter	Value
Total patients	198
Mean age, months, ± SD	36.16 ± 15.08
Gender distribution (male)	53.03%
Primary pathology (comorbidities)	
Hematological	8.58%
Cardiovascular disorders	8.08%
Neurological disorders	17.8%
Respiratory disorders	28.3%
Gastrointestinal disorders	26.8%
Endocrine disorders	1.7%
Shock	8%
Nutritional status	
Malnourished	18.7%
Normal	81.3%
Overnourished	0%

ROC analysis

**Figure 2:**
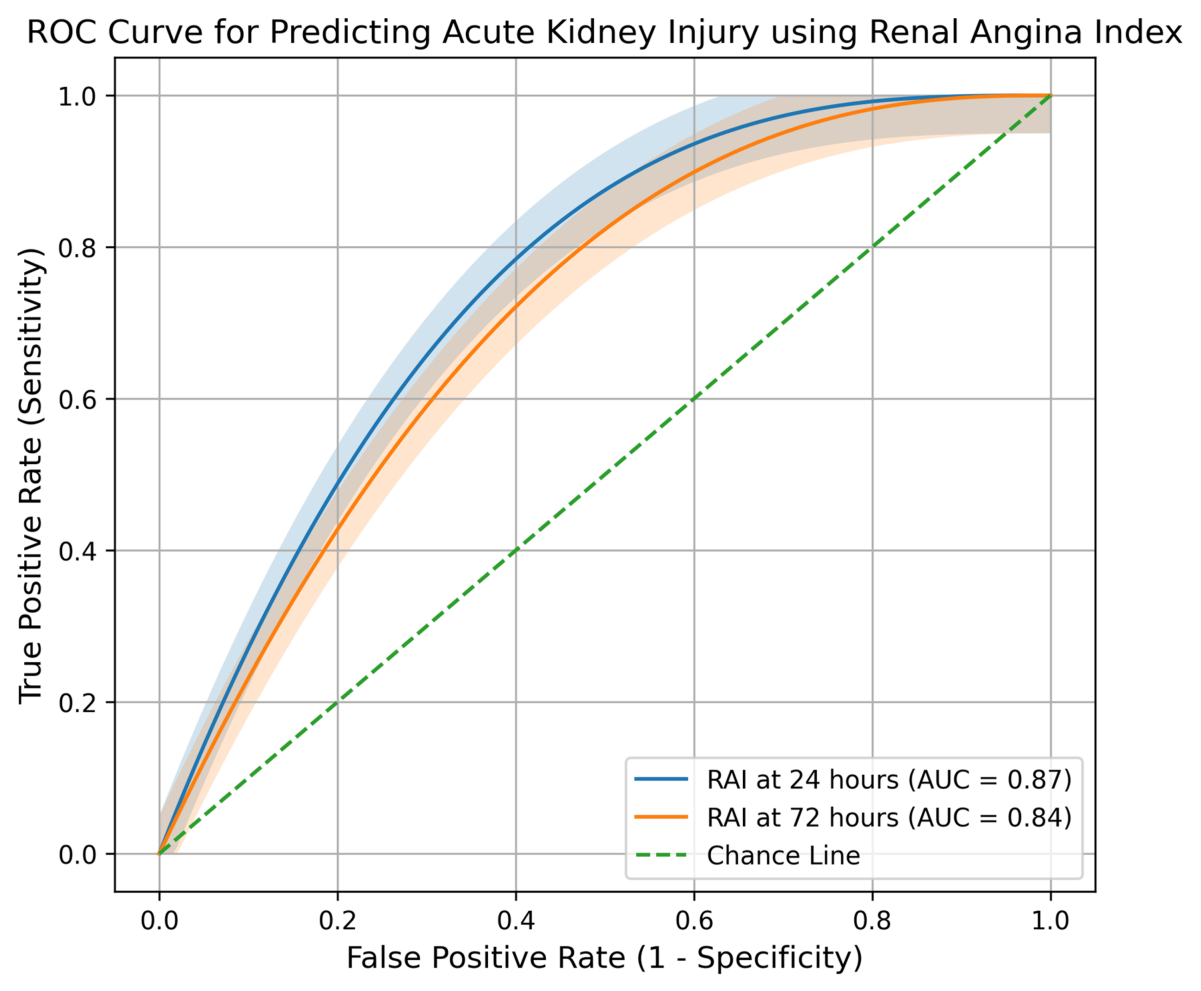
ROC curve comparing RAI at 24 hours (AUC = 0.87) and 72 hours (AUC = 0.84) ROC: receiver operating characteristic; RAI: Renal Angina Index; AUC: area under the curve

RAI demonstrated excellent discriminatory ability for predicting AKI at both measurement timepoints. The 24-hour assessment showed an AUC of 0.87 (95% CI: 0.80-0.94) compared to the 72-hour assessment AUC of 0.84 (95% CI: 0.75-0.93). This difference was not statistically significant (p = 0.412). At 24 hours, RAI threshold ≥ 5 provided 90.9% sensitivity with 79.0% specificity, while threshold ≥ 8 showed 81.8% sensitivity with 97.2% specificity. The higher threshold significantly improved PPV (75.0% vs 27.8%), while NPV remained consistently high (>98%) across all thresholds (Figure 2).

## Discussion

This study demonstrates that RAI has good discriminatory ability for predicting AKI in PICU patients. Our findings revealed an association between RAI scores and mortality, with rates increasing dramatically from 0% in low-risk patients to 68.8% in high-risk categories. This result was similar to that of other studies, which have suggested that patients with more severe disease and higher PRISM scores have higher RAI scores [9,10]. The perfect NPV of low RAI scores for mortality (100%) suggests its potential utility as a rule-out tool, a finding that is consistent with a study by Sundarraju et al. [11].

We observed no KDIGO-defined AKI in patients with low RAI scores, compared to 12.5% in the moderate-risk group and 62.5% in the high-risk group. This gradient indicates that RAI may detect kidney dysfunction before it manifests as clinically apparent AKI according to KDIGO criteria. This finding aligns with those of other studies [12,13] that demonstrated early changes in kidney function biomarkers can predict adverse outcomes in critically ill children. RAI at 24 hours showed slightly better performance (AUC 0.87) than at 72 hours (AUC 0.84), though this difference was not statistically significant. The high sensitivity (90.9%) and NPV (99.0%) of RAI ≥ 5 at 24 hours for predicting AKI suggest its utility for early risk stratification. This early predictive capability provides a valuable window for preventive interventions before kidney damage progresses, consistent with findings by Kaddourah et al. [14], who found that early assessment provided the best opportunity for intervention.

Several clinical parameters showed strong associations with RAI categories. Vasopressor requirements demonstrated a clear gradient across categories (p < 0.001), aligning with the findings of Fitzgerald et al. [15], who identified hemodynamic instability as a key contributor to kidney injury. Similarly, PRISM scores, extended PICU stays, and nephrotoxic medication use all showed significant associations (p < 0.001). The association between RAI and overall illness severity (PRISM scores) supports RAI's relationship with critical illness severity. This suggests that kidney dysfunction captured by RAI represents an important component of overall critical illness in pediatric patients. Neither demographic factors nor most affected organ systems showed significant associations with RAI categorization, suggesting that RAI assesses kidney function relatively independently of these factors.

The emerging paradigm described by Chawla et al. [16] emphasizes the importance of RAI as a tool for the diagnosis of subclinical AKI in critically ill patients, although we were not able to establish such a relationship in our study. The robust performance of RAI at 24 hours has important clinical implications. The excellent rule-out capability (NPV > 98%) could help identify patients at minimal risk, potentially avoiding unnecessary nephroprotective measures. Conversely, the higher PPV of RAI ≥ 8 (75.0% at 24 hours) allows for more precise identification of high-risk patients who may benefit from targeted interventions.

Limitations of the study 

This study has several limitations. Firstly, the single-center design may limit the generalizability of our findings to other settings or populations. Secondly, the relatively small sample size and the lack of multivariate analysis may have affected the robustness of our conclusions. Additionally, the study did not account for potential confounding factors, such as variations in treatment protocols or the presence of other comorbid conditions that could influence outcomes.

## Conclusions

Based on our findings, RAI demonstrates excellent ability to predict both AKI and mortality in the pediatric intensive care setting. The clinically significant predictive value at 24 hours post-admission provides a valuable early window for clinical intervention, while a high NPV makes it particularly useful as a rule-out tool. These findings support the integration of RAI into risk stratification algorithms for critically ill pediatric patients to facilitate earlier recognition and management of kidney injury.
